# Dietary Polyphenols in Aging: A Systems-Level Perspective on Mitochondrial Quality Control and Microbiome Interactions

**DOI:** 10.3390/ijms27093930

**Published:** 2026-04-28

**Authors:** Adnan Yılmaz, Hae-Jin Park, Eun-Mi Ahn, Jaehoon Bae

**Affiliations:** 1 Department of Biochemistry, Faculty of Medicine, Recep Tayyip Erdogan University, 53100 Rize, Türkiye; adnan.yilmaz@erdogan.edu.tr; 2 Department of Foodcare YAKSUN, Daegu Haany University, Gyeongsan-si 38610, Republic of Korea; hjpark@dhu.ac.kr; 3 Functional Food Research Institute, Industry-University Cooperation Foundation, Daegu Haany University, Gyeongsan-si 38610, Republic of Korea; ahnem@dhu.ac.kr; 4 Department of Food Science and Biotechnology, Daegu Haany University, Gyeongsan-si 38610, Republic of Korea; 5 Department of Korea Medical Science, Daegu Haany University, Gyeongsan-si 38610, Republic of Korea

**Keywords:** aging, polyphenols, mitochondrial quality control, mitophagy, cellular senescence, inflammaging, AMPK, SIRT1, Nrf2, gut microbiome

## Abstract

Aging is a multifactorial biological process characterized by progressive functional decline and increased susceptibility to chronic diseases. Targeting the molecular mechanisms underlying aging has therefore emerged as an important strategy for promoting healthy aging. Natural polyphenols, widely present in fruits, vegetables, tea, and medical and aromatic plants, have attracted considerable attention due to their geroprotective properties. This review examines current evidence on the ability of major dietary polyphenols, including resveratrol, epigallocatechin gallate (EGCG), curcumin, and quercetin, to modulate the hallmarks of aging, with particular emphasis on mitochondrial quality control as a central regulatory mechanism. Evidence indicates that polyphenols regulate key signaling pathways involved in aging biology, including AMP-activated protein kinase (AMPK), sirtuins (SIRT), mechanistic target of rapamycin (mTOR), nuclear factor erythroid 2-related factor 2 (Nrf2), and nuclear factor-κB (NF-κB). Through coordinated modulation of these pathways, polyphenols influence mitochondrial biogenesis, mitophagy, redox homeostasis, cellular senescence, and chronic inflammation. In addition, interactions between dietary polyphenols and the gut microbiome generate bioactive metabolites, such as urolithin A, which further contribute to mitochondrial regulation. Overall, polyphenols represent promising modulators of aging-associated pathways and may support strategies aimed at improving healthspan and reducing age-related disease risk.

## 1. Introduction

Population aging represents one of the most significant demographic transformations of the 21st century [1]. Improvements in medical care, nutrition, and living conditions have contributed to substantial increases in life expectancy worldwide [2]. However, increased longevity has also been accompanied by a growing prevalence of chronic age-related diseases, including cardiovascular diseases, neurodegenerative disorders, metabolic syndromes, cancer, and musculoskeletal degeneration [3]. These conditions collectively impose major health, social, and economic burdens on aging societies [4]. Consequently, understanding the biological mechanisms underlying aging has become a central objective in biomedical research [5].

Aging is not a single process but rather a complex biological phenomenon involving progressive deterioration of multiple cellular and physiological systems [6]. At the molecular level, aging is driven by a network of interconnected mechanisms that gradually impair cellular homeostasis [7]. To conceptualize these processes, López-Otín and colleagues proposed the widely recognized framework of the hallmarks of aging [8], which includes genomic instability, telomere attrition, epigenetic alterations, loss of proteostasis, deregulated nutrient sensing, mitochondrial dysfunction, cellular senescence, stem cell exhaustion, and altered intercellular communication. These hallmarks represent key biological processes that collectively contribute to functional decline and increased disease susceptibility during aging. Among these mechanisms, mitochondrial dysfunction, oxidative stress, and chronic inflammation have emerged as central drivers of aging-related pathology [9,10]. Mitochondria play essential roles in cellular energy production, metabolic regulation, and apoptosis [11]. With aging, mitochondrial efficiency declines, leading to increased production of reactive oxygen species (ROS) and impaired ATP generation [12]. Elevated ROS levels can damage cellular macromolecules such as DNA, proteins, and lipids, thereby accelerating cellular dysfunction [13]. Furthermore, oxidative stress can activate inflammatory pathways and promote cellular senescence, contributing to a vicious cycle of tissue degeneration [14].

Cellular senescence is another key hallmark of aging and refers to a stable cell-cycle arrest that occurs in response to various forms of cellular stress [15]. Senescent cells accumulate in aging tissues and secrete a variety of pro-inflammatory factors collectively known as the senescence-associated secretory phenotype (SASP) [16]. These secreted molecules can disrupt tissue structure, impair stem cell function, and promote chronic inflammation, a phenomenon often referred to as inflammaging [17]. Accumulation of senescent cells has been linked to numerous age-related diseases, including osteoarthritis, atherosclerosis, and neurodegenerative disorders [18]. Given the complexity of aging mechanisms, therapeutic strategies targeting a single molecular pathway are unlikely to produce substantial benefits [19]. Instead, interventions that simultaneously influence multiple aging pathways may offer more effective approaches for promoting healthy aging [5,19]. In this context, naturally occurring bioactive compounds have attracted considerable interest due to their ability to modulate diverse cellular signaling networks [20]. Among these compounds, polyphenols represent one of the most extensively studied classes of natural products with potential anti-aging properties [21]. Polyphenols are plant-derived secondary metabolites widely present in fruits, vegetables, tea, cocoa, and medicinal herbs [22]. Polyphenols are traditionally described as antioxidants; however, their biological effects are increasingly understood to involve modulation of redox-sensitive signaling pathways rather than direct radical scavenging alone [23]. Importantly, polyphenols can interact with multiple signaling pathways involved in aging biology [24].

**Figure 1:**
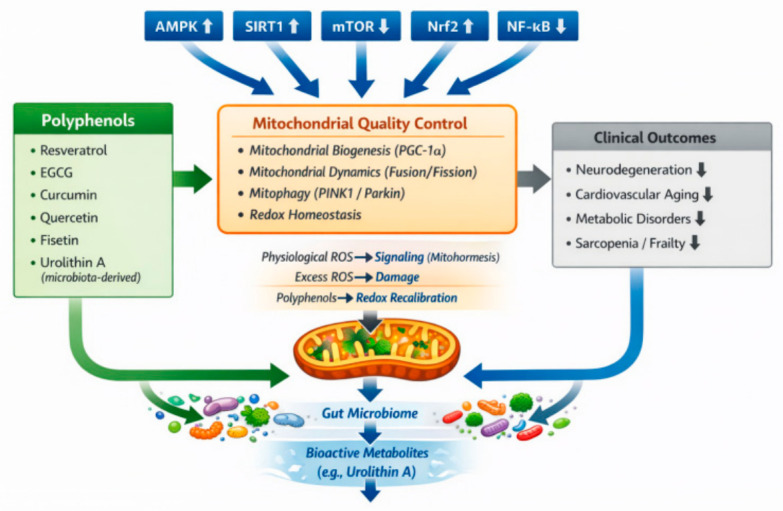
Polyphenols targeting mitochondrial quality control and hallmarks of aging: an integrated mechanistic framework. Dietary polyphenols, including resveratrol, EGCG, curcumin, and quercetin, modulate key signaling pathways involved in aging biology, such as AMPK, SIRT1, mTOR, Nrf2, and NF-κB. These pathways converge on mitochondrial quality control processes, including mitochondrial biogenesis, fusion–fission dynamics, mitophagy, and redox homeostasis. Through coordinated regulation of these mechanisms, polyphenols influence multiple hallmarks of aging, including mitochondrial dysfunction, cellular senescence, oxidative stress, and inflammaging. In addition, gut microbiome-mediated biotransformation of polyphenols generates bioactive metabolites, such as urolithin A, which further enhance mitophagy and mitochondrial function. Collectively, these integrated effects contribute to improved cellular resilience and may attenuate the progression of age-related conditions, including neurodegenerative diseases, cardiovascular dysfunction, metabolic disorders, and frailty.

Several well-known polyphenols have been investigated for their potential geroprotective effects. Resveratrol has been shown to activate sirtuin signaling pathways and mimic aspects of caloric restriction [25]. EGCG exhibits antioxidant and anti-inflammatory properties and modulates mitochondrial function [26]. Curcumin is widely recognized for its anti-inflammatory and cytoprotective activities [27]. Quercetin has attracted attention as a potential senolytic compound capable of reducing the burden of senescent cells [28]. In addition to their direct molecular effects, polyphenols may exert systemic benefits through interactions with the gut microbiome [29]. A substantial proportion of dietary polyphenols reach the colon, where they undergo extensive biotransformation by gut microorganisms [30]. These microbial processes generate metabolites with enhanced bioavailability and biological activity [31]. For example, microbial metabolism of ellagitannins produces urolithin A, which stimulates mitophagy and improves mitochondrial function in aging tissues [32]. The ability of polyphenols to influence multiple biological pathways suggests that these compounds may act as geroprotective agents capable of targeting the fundamental mechanisms of aging [21,24]. However, despite growing interest, a comprehensive understanding of the molecular mechanisms underlying polyphenol-mediated modulation of aging processes remains incomplete [33]. Despite the growing body of literature, most existing reviews have focused primarily on antioxidant properties or individual compounds in isolation [21,33]. However, aging is a systems-level process driven by interconnected mechanisms rather than single molecular events [7,8]. In this context, an integrated framework linking polyphenols to core regulatory networks of aging remains insufficiently developed. The present review addresses this gap by emphasizing mitochondrial quality control and microbiome-mediated metabolism as central mechanisms. A conceptual overview of these interactions is illustrated in Figure 1. While this systems-level perspective provides an integrative framework, it also presents challenges in balancing breadth and mechanistic depth. Therefore, this review aims to provide a conceptual synthesis of key regulatory networks linking polyphenols, mitochondrial quality control, and microbiome interactions, rather than an exhaustive mechanistic or systematic analysis. This review is narrative in nature and does not follow a formal systematic review methodology (e.g., PRISMA), but instead aims to provide an integrative conceptual perspective on the topic.

## 2. Hallmarks of Aging

Aging is a complex biological process driven by the progressive accumulation of cellular damage and dysregulation of multiple molecular pathways [6]. To conceptualize these processes, López-Otín and colleagues proposed the widely accepted framework of the hallmarks of aging [8], which describes several interconnected biological processes that contribute to functional decline and increased vulnerability to disease during aging. Recent updates to the hallmarks of aging framework have expanded this model to include additional features such as chronic inflammation, dysbiosis, and impaired macroautophagy, reflecting a more integrated and systems-level understanding of aging biology [10,34]. These hallmarks include genomic instability, telomere attrition, epigenetic alterations, loss of proteostasis, deregulated nutrient sensing, mitochondrial dysfunction, cellular senescence, stem cell exhaustion, and altered intercellular communication. Although each hallmark represents a distinct biological mechanism, they are highly interconnected and collectively contribute to the aging phenotype.

### 2.1. Genome Instability, Telomere Attrition, and Epigenetic Alterations

Genomic instability represents one of the earliest events associated with aging [35]. Cells are continuously exposed to endogenous and exogenous factors capable of inducing DNA damage, including oxidative stress, replication errors, radiation, and environmental toxins. Accumulation of DNA lesions over time can lead to mutations, chromosomal rearrangements, and impaired gene expression [36]. Although cells possess multiple DNA repair mechanisms, the efficiency of these systems declines with age, resulting in progressive genomic instability [37]. DNA damage can activate stress responses that promote senescence, apoptosis, or metabolic reprogramming, thereby contributing to tissue dysfunction and aging-related pathologies. Closely related to genomic instability is telomere attrition. Telomeres progressively shorten with each cell division and eventually trigger DNA damage responses when reaching a critical length [38]. Shortened telomeres activate p53-dependent pathways that lead to cell-cycle arrest or apoptosis and have been associated with numerous age-related diseases [39]. Epigenetic alterations further contribute to aging. These include DNA methylation, histone modifications, and chromatin remodeling [40]. Age-associated epigenetic drift affects gene expression patterns involved in metabolism, inflammation, and stress responses [41]. The development of epigenetic clocks has provided important insights into biological aging and disease susceptibility [42].

### 2.2. Proteostasis Decline and Autophagy Dysfunction

The loss of proteostasis is a key feature of aging [43]. Proteostasis involves the coordinated regulation of protein synthesis, folding, trafficking, and degradation. With aging, these systems become compromised, leading to the accumulation of misfolded or damaged proteins [44]. This is particularly evident in neurodegenerative diseases, where protein aggregates contribute to disease pathology [45]. Closely related to proteostasis is the decline of autophagy, a cellular recycling process that removes damaged proteins and organelles [46]. Reduced autophagic activity during aging leads to the accumulation of dysfunctional cellular components and contributes to mitochondrial dysfunction, oxidative stress, and increased disease susceptibility [47]. Enhancing autophagy has therefore emerged as a promising strategy for promoting healthy aging.

### 2.3. Mitochondrial Dysfunction, Cellular Senescence, and Inflammaging

Mitochondrial dysfunction is a central hallmark of aging [9,12]. Mitochondria are responsible for ATP production and metabolic regulation but also generate ROS as by-products of oxidative phosphorylation. During aging, mitochondrial DNA mutations accumulate, respiratory efficiency declines, and ROS production increases [13]. Elevated ROS levels can damage DNA, proteins, and lipids and activate inflammatory signaling pathways [14]. In addition, mitochondrial quality control mechanisms such as mitophagy become impaired, allowing dysfunctional mitochondria to accumulate [9]. These processes contribute to metabolic dysregulation and cellular decline. Cellular senescence is characterized by irreversible cell-cycle arrest in response to stress signals such as DNA damage, oxidative stress, and mitochondrial dysfunction [15]. Senescent cells remain metabolically active but exhibit profound changes in gene expression and secretory activity. A defining feature of senescence is the SASP [16], which includes pro-inflammatory cytokines, growth factors, and proteases. SASP factors disrupt tissue structure, impair stem cell function, and promote chronic inflammation [17]. This persistent inflammatory state, known as inflammaging, arises from multiple sources including senescent cells, immune dysregulation, and mitochondrial dysfunction [17]. Inflammaging plays a central role in the progression of age-related diseases.

### 2.4. Nutrient Sensing, Stem Cell Exhaustion, and Intercellular Communication

Aging is also characterized by deregulated nutrient sensing [7]. Key pathways include insulin/IGF-1 signaling, AMPK, sirtuins, and mTOR. These pathways regulate energy metabolism and cellular growth. Dysregulation of these systems contributes to metabolic disorders and reduced stress resilience. Stem cell exhaustion represents another hallmark of aging [48]. With age, stem cells lose their regenerative capacity, leading to impaired tissue repair and functional decline across multiple organs [49]. Finally, altered intercellular communication contributes to aging [50]. Changes in inflammatory signaling, immune function, and hormonal regulation disrupt tissue homeostasis. These processes interact with other hallmarks to accelerate aging and disease progression. Although each hallmark contributes individually to aging, these mechanisms are highly interconnected [8]. For example, mitochondrial dysfunction increases oxidative stress, which promotes genomic instability and senescence. Similarly, chronic inflammation exacerbates metabolic dysregulation and tissue degeneration. Because aging arises from an interconnected network of processes, interventions targeting multiple hallmarks simultaneously may provide the most effective strategy for delaying aging and preventing disease [19]. In this context, dietary polyphenols have attracted increasing attention as potential modulators of aging-associated pathways [21]. Polyphenols have been reported to modulate mitochondrial function, oxidative stress, and cellular senescence, primarily based on evidence from in vitro and animal studies, while clinical validation remains limited. The following sections examine how these compounds interact with these mechanisms and their potential as geroprotective agents.

## 3. Major Polyphenols with Anti-Aging Activity

Natural polyphenols represent one of the most extensively investigated classes of dietary phytochemicals with potential geroprotective properties [21,33]. Polyphenols are widely distributed in fruits, vegetables, tea, coffee, cocoa, and medicinal plants and exhibit diverse biological activities including antioxidant, anti-inflammatory, metabolic regulatory, and cytoprotective effects [22,23,24]. In the context of aging biology, these compounds are particularly interesting because they modulate multiple signaling pathways associated with the hallmarks of aging [21,24]. Among the numerous polyphenols investigated, resveratrol, epigallocatechin gallate (EGCG), curcumin, and quercetin are the most extensively studied compounds due to their ability to regulate several longevity-associated pathways, including AMPK, SIRT1, mTOR, Nrf2, and NF-κB signaling [21,33]. Where available, primary experimental studies have been preferentially considered to support mechanistic interpretations, in order to reduce potential bias associated with reliance on secondary sources.

### 3.1. Resveratrol

Resveratrol (3,5,4′-trihydroxystilbene) is a naturally occurring stilbene polyphenol found in grapes, berries, peanuts, and red wine [25]. It has attracted significant scientific interest due to its potential role in the so-called “French paradox,” where populations consuming diets rich in red wine exhibit relatively low rates of cardiovascular disease despite high dietary fat intake [51]. Since the discovery that resveratrol activates sirtuin signaling pathways, it has become one of the most widely studied compounds in aging research [52]. One of the primary mechanisms underlying the anti-aging effects of resveratrol involves the activation of SIRT1, a NAD^+^-dependent deacetylase that regulates numerous transcription factors involved in metabolism, stress resistance, and mitochondrial function [25,52]. Activation of SIRT1 by resveratrol leads to deacetylation of the transcriptional coactivator PGC-1α, which promotes mitochondrial biogenesis and oxidative metabolism [53]. Increased mitochondrial biogenesis improves energy production and reduces mitochondrial dysfunction, a central hallmark of aging [53,54].

Resveratrol also activates AMPK, a critical cellular energy sensor that regulates metabolic homeostasis [54,55]. Activation of AMPK promotes fatty acid oxidation, enhances glucose uptake, and stimulates autophagy. Through AMPK activation, resveratrol indirectly suppresses the mTOR pathway, which is known to promote anabolic growth and inhibit autophagy [56]. Inhibition of mTOR signaling is associated with enhanced cellular maintenance and increased lifespan in several model organisms [56].

Another important aspect of resveratrol biology is its capacity to reduce oxidative stress and inflammation. Resveratrol can activate Nrf2 signaling, leading to the transcription of antioxidant enzymes such as heme oxygenase-1 (HO-1), glutathione peroxidase, and superoxide dismutase [25,57]. In addition, resveratrol suppresses NF-κB signaling, thereby reducing the expression of pro-inflammatory cytokines that contribute to inflammaging [25,54]. Through these combined mechanisms, resveratrol modulates mitochondrial function, oxidative stress responses, and inflammatory signaling networks associated with aging.

### 3.2. Epigallocatechin Gallate (EGCG)

Epigallocatechin gallate (EGCG) is the major catechin found in green tea and is responsible for many of the health benefits associated with tea consumption [26,58]. EGCG has been shown to exert antioxidant, anti-inflammatory, and metabolic regulatory effects, making it an important compound in aging research [26,58]. One of the key mechanisms of EGCG action involves the activation of Nrf2-mediated antioxidant signaling [26,57]. Nrf2 is a transcription factor that regulates the expression of numerous antioxidant and detoxification enzymes. Activation of Nrf2 leads to increased expression of genes involved in glutathione metabolism, ROS detoxification, and cellular stress resistance. By enhancing the endogenous antioxidant defense system, EGCG can reduce oxidative damage to DNA, proteins, and lipids [26,58]. EGCG also influences mitochondrial function and energy metabolism. Studies have shown that EGCG can enhance mitochondrial respiration and improve mitochondrial membrane potential, thereby supporting efficient oxidative phosphorylation [58,59]. These effects are partly mediated through AMPK activation, which promotes metabolic adaptation during cellular stress [58]. In addition to its metabolic effects, EGCG has been shown to regulate inflammatory pathways. EGCG can inhibit activation of NF-κB signaling, thereby reducing the expression of pro-inflammatory cytokines such as interleukin-6 (IL-6), tumor necrosis factor-α (TNF-α), and IL-1β [26,60]. Because chronic inflammation is a key feature of aging and contributes to many age-related diseases, suppression of inflammatory signaling by EGCG may play an important role in its anti-aging activity.

### 3.3. Curcumin

Curcumin is a polyphenolic compound derived from the rhizome of Curcuma longa, commonly known as turmeric [27]. It has been used for centuries in traditional medicine and has gained significant attention in modern biomedical research due to its potent anti-inflammatory and antioxidant properties [27,61]. Curcumin exerts its biological effects through modulation of several signaling pathways involved in aging biology. One of the most well-characterized mechanisms involves inhibition of NF-κB signaling, which plays a central role in the regulation of inflammatory responses [27,61]. By suppressing NF-κB activation, curcumin reduces the expression of inflammatory cytokines and mediators that contribute to inflammaging [27,61]. Curcumin also influences Nrf2 signaling, which enhances cellular antioxidant defenses and protects cells from oxidative damage [59]. Activation of Nrf2 leads to increased transcription of genes encoding detoxification enzymes and antioxidant proteins. These mechanisms help maintain redox balance and protect against oxidative stress-induced cellular damage [59]. Another important mechanism involves the regulation of autophagy, a cellular recycling process that removes damaged proteins and organelles. Curcumin has been reported to induce autophagy through modulation of the AMPK–mTOR signaling axis [62]. By promoting autophagic activity, curcumin helps maintain proteostasis and prevents the accumulation of dysfunctional cellular components. Despite these promising biological effects, curcumin faces several challenges in clinical applications due to its low bioavailability and rapid metabolism [27]. Nevertheless, advances in drug delivery systems, including nanoparticle formulations and liposomal encapsulation, may improve its pharmacokinetic properties and enhance its therapeutic potential [27].

### 3.4. Quercetin

Quercetin is a flavonoid widely found in fruits, vegetables, and plant-derived foods such as apples, onions, berries, and capers [63]. It possesses strong antioxidant and anti-inflammatory properties and has recently attracted attention as a potential senolytic compound [28,63]. Senolytics are agents capable of selectively eliminating senescent cells. Senescent cells accumulate in aging tissues and contribute to chronic inflammation through the secretion of SASP factors. Quercetin has been shown to reduce the burden of senescent cells by targeting pro-survival signaling pathways that allow senescent cells to persist [28]. These pathways include PI3K/Akt signaling and anti-apoptotic networks [28]. In addition to its senolytic activity, quercetin also exerts antioxidant and anti-inflammatory effects. Quercetin can directly scavenge reactive oxygen species and inhibit inflammatory signaling pathways, including NF-κB activation [63]. Through these mechanisms, quercetin may reduce oxidative stress and suppress SASP-associated inflammatory responses. Another interesting aspect of quercetin biology is its potential role in modulating mitochondrial function. Some studies suggest that quercetin may enhance mitochondrial biogenesis and improve mitochondrial respiration, although the precise molecular mechanisms remain under investigation [64]. 

Taken together, the major polyphenols discussed in this section share several common mechanisms of action despite their structural diversity. These compounds converge on key signaling pathways involved in energy metabolism, oxidative stress responses, inflammation, and cellular maintenance [21,24,33]. Through the coordinated regulation of these pathways, polyphenols may influence multiple hallmarks of aging and contribute to improved cellular resilience. It is important to note that much of the evidence supporting these effects derives from cellular and animal models, and the extent to which these findings translate into clinically meaningful outcomes in humans remains uncertain [Table 1].

## 4. Molecular Signaling Pathways Regulating Aging

The biological effects of polyphenols on aging are largely mediated through the regulation of several conserved cellular signaling pathways that coordinate metabolic homeostasis, stress resistance, and cellular maintenance [81,82]. These signaling pathways do not operate in isolation but rather function within highly interconnected and context-dependent regulatory networks. Therefore, their modulation by polyphenols should be interpreted within a systems-level framework rather than as linear cause–effect relationships. Among these pathways, AMPK, sirtuins (SIRT), mechanistic target of rapamycin (mTOR), nuclear factor erythroid 2–related factor 2 (Nrf2), and nuclear factor kappa B (NF-κB) are particularly important because they integrate nutrient sensing, redox balance, mitochondrial function, autophagy, and inflammatory responses [81,82,83,84]. Dysregulation of these pathways contributes to many hallmarks of aging, whereas pharmacological or nutritional modulation of these pathways may delay aging-related cellular decline. Accordingly, modulation of these pathways by polyphenols should not be directly equated with systemic anti-aging effects, particularly in the absence of robust clinical evidence.

### 4.1. AMP-Activated Protein Kinase (AMPK)

AMPK is a central energy sensor that maintains cellular energy homeostasis [81,85]. AMPK is activated when intracellular energy levels decline, resulting in increased AMP/ATP or ADP/ATP ratios. Upon activation, AMPK promotes catabolic processes that generate ATP while inhibiting energy-consuming anabolic pathways [81,85]. AMPK plays a key role in aging biology because it regulates several processes associated with longevity, including mitochondrial biogenesis, autophagy, and metabolic adaptation [81,85]. Activation of AMPK stimulates the transcriptional coactivator PGC-1α, which promotes mitochondrial biogenesis and enhances oxidative metabolism [54,85]. Increased mitochondrial biogenesis improves cellular energy production and reduces the accumulation of dysfunctional mitochondria [54]. Polyphenols have been reported to activate AMPK signaling in various cellular and animal models [81]. Resveratrol, for example, stimulates AMPK activation indirectly through changes in cellular energy metabolism and SIRT1 activation [54]. EGCG and quercetin have also been shown to enhance AMPK activity, contributing to improved mitochondrial function and metabolic homeostasis [58,64]. Through these mechanisms, AMPK activation by polyphenols may enhance cellular stress resistance and promote longevity-associated phenotypes. Another important role of AMPK is the regulation of autophagy, a cellular process that degrades damaged proteins and organelles. AMPK activates autophagy by inhibiting mTOR signaling and directly phosphorylating autophagy-related proteins such as ULK1 [81,85]. Because impaired autophagy is a hallmark of aging, activation of AMPK represents an important mechanism through which polyphenols may promote cellular maintenance and longevity.

### 4.2. Sirtuin Signaling

Sirtuins are a family of NAD^+^-dependent deacetylases that regulate numerous biological processes, including metabolism, stress responses, DNA repair, and mitochondrial function [86]. Among the seven mammalian sirtuins, SIRT1 has been the most extensively studied in the context of aging [86]. SIRT1 regulates cellular metabolism and stress resistance through deacetylation of multiple transcription factors and co-regulators. One of the most important targets of SIRT1 is PGC-1α, which controls mitochondrial biogenesis and oxidative metabolism [53,86]. Deacetylation of PGC-1α by SIRT1 enhances its transcriptional activity, leading to increased expression of genes involved in mitochondrial function and energy metabolism [53,86]. SIRT1 also influences cellular survival pathways by modulating transcription factors such as FOXO, p53, and NF-κB [86]. Deacetylation of FOXO proteins promotes the expression of genes involved in antioxidant defense and stress resistance. At the same time, SIRT1-mediated regulation of NF-κB signaling reduces inflammatory responses that contribute to aging-related tissue dysfunction [86]. Resveratrol is widely recognized as one of the most prominent activators of SIRT1 signaling [25,52]. Through activation of SIRT1, resveratrol enhances mitochondrial function, improves metabolic regulation, and promotes cellular stress resistance [53,54]. Although the precise molecular mechanism by which resveratrol activates SIRT1 remains debated, numerous studies have demonstrated that resveratrol-induced SIRT1 activation contributes to improved mitochondrial homeostasis and longevity-associated phenotypes [25,52,53,54].

### 4.3. Mechanistic Target of Rapamycin (mTOR)

The mechanistic target of rapamycin (mTOR) is a highly conserved serine/threonine kinase that regulates cellular growth, metabolism, and protein synthesis [82,85]. mTOR functions within two distinct complexes, mTORC1 and mTORC2, which respond to nutrient availability, growth factors, and cellular energy status [82]. mTORC1 is particularly important in aging research because excessive activation of this pathway promotes anabolic processes that may contribute to cellular damage accumulation over time [82]. Hyperactivation of mTOR signaling has been associated with reduced autophagy, impaired proteostasis, and metabolic dysregulation [82,85]. Conversely, inhibition of mTOR signaling has been shown to extend lifespan in several model organisms [82,87]. Polyphenols can influence mTOR signaling either directly or indirectly through upstream regulators such as AMPK [81,85]. Activation of AMPK by polyphenols leads to inhibition of mTORC1 activity, thereby promoting autophagy and cellular maintenance processes [81,85]. Curcumin has also been reported to suppress mTOR signaling, which may contribute to its ability to induce autophagy and reduce the accumulation of damaged cellular components [62]. The balance between AMPK activation and mTOR inhibition plays a crucial role in determining cellular responses to metabolic stress. By modulating this balance, polyphenols may enhance cellular resilience and delay aging-related functional decline.

### 4.4. Nrf2-Mediated Antioxidant Defense

The transcription factor Nrf2 (nuclear factor erythroid 2–related factor 2) plays a central role in regulating cellular responses to oxidative stress [83,88]. Under normal conditions, Nrf2 is sequestered in the cytoplasm by its inhibitor Keap1 and targeted for degradation. However, during oxidative stress, Nrf2 dissociates from Keap1 and translocates to the nucleus, where it activates the transcription of genes encoding antioxidant and detoxification enzymes [83,88]. Nrf2-regulated genes include heme oxygenase-1 (HO-1), glutathione peroxidase, superoxide dismutase, and NAD(P)H quinone oxidoreductase. These enzymes contribute to the detoxification of reactive oxygen species and help maintain redox homeostasis [83,88]. Polyphenols such as EGCG and curcumin have been shown to activate Nrf2 signaling, leading to increased antioxidant defense capacity [57,59]. Activation of the Nrf2 pathway protects cells from oxidative damage and may reduce the accumulation of DNA mutations, protein damage, and lipid peroxidation associated with aging [83,88]. Because oxidative stress contributes to multiple hallmarks of aging—including mitochondrial dysfunction, genomic instability, and cellular senescence—activation of Nrf2 represents an important mechanism through which polyphenols may exert anti-aging effects.

### 4.5. NF-κB and Inflammatory Signaling

Chronic inflammation is a major driver of aging and age-related diseases [10,17,84]. One of the key regulators of inflammatory responses is the transcription factor NF-κB (nuclear factor kappa B) [84,89]. NF-κB controls the expression of numerous inflammatory mediators, including cytokines, chemokines, and adhesion molecules [89]. In aging tissues, persistent activation of NF-κB signaling contributes to the development of inflammaging, a state of chronic low-grade inflammation that promotes tissue degeneration and disease progression [10,17,84]. NF-κB signaling also plays a central role in the regulation of the SASP, which amplifies inflammatory signaling in aging tissues [16,17,84,90]. Polyphenols have been shown to inhibit NF-κB signaling in various experimental models [21,33]. Curcumin is particularly well known for its ability to suppress NF-κB activation by inhibiting upstream signaling pathways involved in inflammatory responses [61,91]. Resveratrol and quercetin have also been reported to attenuate NF-κB signaling, thereby reducing inflammatory cytokine production [25,63,92]. Through suppression of inflammatory signaling, polyphenols may reduce the chronic inflammation associated with aging and help maintain tissue homeostasis.

## 5. Polyphenols and Cellular Senescence

**Figure 2:**
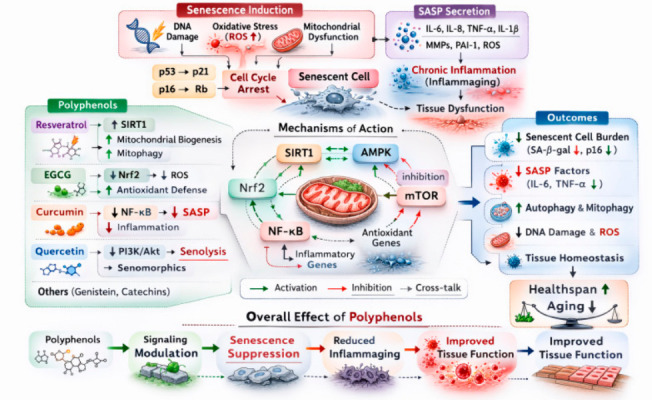
Polyphenol-mediated modulation of cellular senescence and SASP. Cellular senescence is induced by stressors such as DNA damage, oxidative stress, and mitochondrial dysfunction, leading to activation of p53–p21 and p16–Rb pathways and irreversible cell-cycle arrest. Senescent cells acquire a SASP, characterized by the release of pro-inflammatory cytokines, chemokines, and proteases that contribute to chronic inflammation (inflammaging) and tissue dysfunction. Polyphenols modulate senescence-associated pathways through multiple mechanisms. Resveratrol activates SIRT1-dependent signaling and supports mitochondrial function, EGCG enhances Nrf2-mediated antioxidant responses, curcumin suppresses NF-κB–dependent inflammatory signaling, and quercetin exerts senolytic and senomorphic effects by targeting pro-survival pathways such as PI3K/Akt. Through these coordinated actions, polyphenols attenuate senescent cell burden, reduce SASP-associated inflammation, and promote tissue homeostasis during aging.

An overview of polyphenol-mediated modulation of cellular senescence is illustrated in Figure 2.

Cellular senescence is a fundamental hallmark of aging characterized by a stable and essentially irreversible arrest of the cell cycle accompanied by profound alterations in gene expression, metabolism, chromatin organization, and secretory activity [93]. Senescence can be induced by a wide range of cellular stressors, including telomere shortening, oxidative stress, oncogenic signaling, DNA damage, mitochondrial dysfunction, and metabolic imbalance [93,94]. Although senescence initially functions as a protective mechanism that prevents the proliferation of damaged or potentially oncogenic cells, the progressive accumulation of senescent cells in aging tissues contributes to chronic inflammation, impaired tissue regeneration, and age-related functional decline [95]. One of the defining features of senescent cells is the SASP [96]. SASP involves the secretion of numerous pro-inflammatory cytokines, chemokines, growth factors, and proteases, including IL-6, interleukin-8 (IL-8), TNF-α, matrix metalloproteinases (MMPs), and various growth regulators. These secreted factors profoundly influence the surrounding tissue microenvironment and may propagate senescence in neighboring cells through paracrine signaling [96,97]. Consequently, SASP contributes to tissue dysfunction, stem cell impairment, and chronic inflammation, collectively promoting the phenomenon known as inflammaging [97].

The molecular regulation of cellular senescence involves several interconnected signaling pathways, among which the p53–p21 and p16–Rb pathways are considered the central regulators of cell-cycle arrest [94,98]. DNA damage responses activate the tumor suppressor protein p53, which subsequently induces expression of the cyclin-dependent kinase inhibitor p21. Elevated p21 levels inhibit cyclin-dependent kinases and prevent phosphorylation of retinoblastoma protein (Rb), thereby blocking progression through the G1/S phase of the cell cycle. In parallel, activation of the p16 pathway also inhibits cyclin-dependent kinases, reinforcing cell-cycle arrest through stabilization of the Rb protein [98]. Persistent activation of these pathways results in irreversible cellular senescence.

Recent research has demonstrated that mitochondrial dysfunction plays a critical role in the initiation and maintenance of senescence [99]. Dysfunctional mitochondria generate excessive levels of reactive oxygen species (ROS), which can cause DNA damage and activate senescence-associated signaling pathways [99,100]. Moreover, impaired mitochondrial quality control leads to accumulation of damaged organelles, further amplifying oxidative stress and inflammatory signaling [100]. These mechanisms illustrate the close interplay between mitochondrial dysfunction, oxidative stress, and senescence.

Natural polyphenols have emerged as important modulators of senescence-associated pathways due to their capacity to regulate oxidative stress, mitochondrial function, and inflammatory signaling [101]. Several polyphenols can attenuate senescence by reducing ROS production and enhancing antioxidant defense mechanisms. For example, resveratrol has been shown to activate SIRT1 signaling, which enhances mitochondrial biogenesis and improves cellular stress resistance [102]. Through activation of SIRT1 and AMPK pathways, resveratrol promotes mitochondrial turnover and enhances autophagy, thereby preventing the accumulation of dysfunctional mitochondria that may trigger senescence [102,103].

In addition to resveratrol, EGCG has also been reported to influence senescence-associated pathways through activation of Nrf2-mediated antioxidant responses [104]. Nrf2 regulates the transcription of multiple antioxidant enzymes that neutralize reactive oxygen species and maintain redox homeostasis. By enhancing the endogenous antioxidant defense system, EGCG may reduce oxidative DNA damage and attenuate the activation of senescence signaling pathways [104]. Furthermore, EGCG has been shown to suppress inflammatory signaling and reduce the expression of pro-inflammatory mediators associated with SASP [105].

Curcumin represents another polyphenol with strong modulatory effects on cellular senescence. Curcumin is known to inhibit NF-κB signaling, which plays a central role in regulating inflammatory responses and SASP production [106]. Persistent activation of NF-κB is commonly observed in aging tissues and contributes to chronic inflammatory signaling. By suppressing NF-κB activation, curcumin may reduce the production of SASP-associated inflammatory cytokines and mitigate the deleterious effects of senescent cells on tissue homeostasis. In addition, curcumin has been reported to influence epigenetic regulation and transcriptional networks involved in stress responses and cellular survival [106].

Among the polyphenols investigated in aging research, quercetin has received particular attention for its senolytic properties [107]. Senolytics are compounds capable of selectively eliminating senescent cells by targeting pro-survival pathways that allow these cells to persist despite cellular damage. Quercetin has been shown to modulate pathways involved in apoptosis resistance, including PI3K/Akt signaling and anti-apoptotic networks [107]. Experimental studies have demonstrated that quercetin can reduce senescent cell burden and improve tissue function in aging models, particularly when used in combination with other senolytic compounds such as dasatinib [107]. In addition to senolytic activity, quercetin may also function as a senomorphic compound, meaning that it reduces the harmful effects of senescent cells without necessarily eliminating them. Senomorphic compounds suppress SASP-associated inflammatory signaling and mitigate tissue damage caused by senescent cells [107]. This distinction between senolytic and senomorphic activities is important because both mechanisms may contribute to improved tissue homeostasis during aging. Another emerging concept in aging biology is that senescence is not solely a cell-autonomous process but is also influenced by systemic factors such as metabolic status, immune signaling, and microbiome composition [108]. Polyphenols may therefore influence senescence indirectly through modulation of these systemic processes. For example, polyphenols can influence gut microbiota composition, leading to the generation of bioactive microbial metabolites that regulate mitochondrial function and inflammation [108].

Overall, polyphenols appear to influence cellular senescence through several interconnected mechanisms, including reduction of oxidative stress, improvement of mitochondrial quality control, suppression of inflammatory signaling, and modulation of apoptosis-resistant pathways [101]. Because cellular senescence represents a central driver of aging and age-related diseases, targeting senescence pathways with polyphenols may provide an effective strategy for promoting healthy aging. However, despite promising results from experimental models, several challenges remain in translating these findings into clinical interventions. Polyphenols often exhibit limited bioavailability, extensive metabolism, and significant inter-individual variability in response due to differences in gut microbiome composition [109]. Future research should therefore focus on improving the pharmacokinetic properties of polyphenols and identifying biomarkers that can predict individual responses to polyphenol-based interventions.

## 6. Polyphenols and Mitochondrial Quality Control

**Figure 3:**
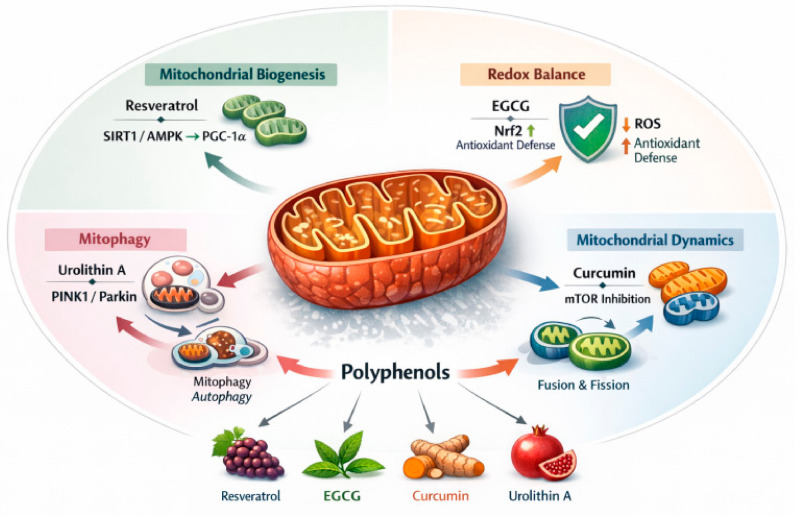
Polyphenol-mediated regulation of mitochondrial quality control processes. Polyphenols modulate mitochondrial quality control through coordinated regulation of mitochondrial biogenesis, dynamics, mitophagy, and redox homeostasis. Resveratrol activates SIRT1- and AMPK-dependent signaling, promoting PGC-1α–mediated mitochondrial biogenesis and metabolic adaptation. Epigallocatechin gallate (EGCG) enhances nuclear factor erythroid 2–related factor 2 (Nrf2)-mediated antioxidant responses, thereby maintaining redox balance and reducing oxidative stress. Curcumin influences mitochondrial dynamics and autophagy-related processes through modulation of mTOR signaling and stress-responsive pathways, contributing to the maintenance of mitochondrial network integrity. In addition, urolithin A, a gut microbiota-derived metabolite, induces PINK1/Parkin-dependent mitophagy, facilitating the selective removal of dysfunctional mitochondria. Through these integrated mechanisms, polyphenols support mitochondrial turnover, preserve redox homeostasis, and enhance cellular resilience under aging-associated stress conditions.

The regulatory effects of polyphenols on mitochondrial quality control processes are summarized in Figure 3. Mitochondrial dysfunction is not merely one hallmark among many in aging biology; rather, it functions as a systems-level hub that connects energy failure, redox imbalance, inflammatory activation, defective proteostasis, and cellular senescence [110,111]. Mitochondria regulate ATP production through oxidative phosphorylation and provide essential biosynthetic intermediates. They also buffer intracellular calcium and participate in apoptotic signaling [112]. Because of these central functions, even modest impairment in mitochondrial homeostasis can propagate across multiple cellular compartments and amplify age-associated decline. Aging mitochondria typically exhibit reduced respiratory efficiency and impaired electron transport chain function. These changes are accompanied by altered membrane potential, increased mitochondrial reactive oxygen species (mtROS) production, accumulation of mitochondrial DNA (mtDNA) lesions, and defective organelle turnover [110,113]. These changes are not independent events. Instead, they reinforce one another and gradually establish a self-perpetuating cycle of mitochondrial damage and cellular dysfunction.

A key concept that has emerged in recent years is that mitochondrial aging is driven not only by primary damage but also by the failure of mitochondrial quality control. Mitochondrial quality control consists of several tightly coordinated processes. These include mitochondrial biogenesis, mitochondrial dynamics (fusion and fission), proteostasis within the organelle, and selective clearance of damaged mitochondria by mitophagy [110,114]. These systems allow the cell to maintain a functional mitochondrial network despite continuous metabolic stress. With aging, however, this network becomes progressively less adaptive. Reduced mitochondrial biogenesis limits the replacement of damaged organelles. Impaired fusion–fission dynamics compromise stress buffering and organelle segregation. In addition, defective mitophagy allows dysfunctional mitochondria to persist. The resulting mtROS excess promotes oxidative damage to mtDNA, mitochondrial proteins, and membrane lipids. This further worsens respiratory dysfunction and contributes to senescence-associated signaling [110,113].

### 6.1. Mitochondrial Biogenesis as a Longevity-Associated Adaptive Program

Mitochondrial biogenesis is the process through which new mitochondria are generated and functionally integrated into the existing network [115]. This process depends on coordinated signaling between the nucleus and mitochondria. It is primarily regulated by PGC-1α, which acts as a transcriptional coactivator for nuclear respiratory factors and other programs controlling oxidative metabolism [115,116]. In aging tissues, PGC-1α activity frequently declines, contributing to reduced respiratory capacity, diminished metabolic flexibility, and impaired adaptation to stress [116]. Because mitochondrial biogenesis is closely coupled to nutrient sensing, it is strongly influenced by AMPK and SIRT1, two pathways repeatedly implicated in longevity and targeted by polyphenols [81,86]. AMPK promotes PGC-1α activation under energy stress, whereas SIRT1 deacetylates PGC-1α and enhances its transcriptional activity [115,116]. Together, these pathways shift the cell toward oxidative metabolism, mitochondrial renewal, and improved energetic resilience.

Within this framework, resveratrol remains the prototypical mitochondrial polyphenol. Although the exact hierarchy of its proximal molecular interactions remains debated, the dominant functional pattern is consistent. Resveratrol enhances AMPK/SIRT1-linked signaling, supports PGC-1α-dependent mitochondrial biogenesis, and improves mitochondrial metabolism under aging-associated stress conditions [102,103,117]. Importantly, this should not be interpreted as a simple increase in mitochondrial number. Rather, resveratrol appears to promote qualitative remodeling of the mitochondrial network—favoring organelles with improved oxidative efficiency, lower ROS leakage, and greater adaptability to energetic challenge. In aging biology, this distinction matters because an expansion of dysfunctional mitochondria would be maladaptive, whereas renewal of a metabolically competent network is protective.

EGCG and quercetin are also linked to mitochondrial biogenic programs, although usually less directly than resveratrol. Their effects appear to depend more heavily on redox-sensitive signaling, especially by limiting oxidative injury that would otherwise suppress mitochondrial function and destabilize mitochondrial proteins and lipids [104,118]. In this sense, some polyphenols may preserve mitochondrial biogenesis not by strongly activating the biogenic machinery per se, but by maintaining the intracellular environment in which biogenesis remains effective. This is a mechanistically important distinction because successful mitochondrial renewal requires both upstream signaling competence and adequate protection from oxidative and inflammatory disruption.

### 6.2. Mitochondrial Dynamics: Fusion, Fission, and Network Integrity

Direct evidence that polyphenols normalize fusion–fission balance remains less extensive than evidence for their effects on AMPK, SIRT1, or Nrf2. However, emerging work suggests that polyphenols may influence mitochondrial dynamics indirectly by reducing oxidative stress, improving membrane potential, and restoring upstream signaling environments that determine whether fusion or fission predominates [114,119]. This indirect route may be particularly relevant in aging, where defects in dynamics are rarely isolated events and more commonly arise from broader changes in cellular stress, metabolic overload, and impaired autophagic competence. Therefore, when polyphenols improve mitochondrial dynamics, the effect is likely to be network-level and context-dependent, rather than the result of a single direct protein target.

### 6.3. Mitophagy as the Critical Elimination Arm of Mitochondrial Quality Control

Among all mitochondrial quality-control mechanisms, mitophagy has become especially important in aging research because it determines whether damaged mitochondria are removed before they become chronic sources of mtROS and inflammatory signaling [120]. The best-characterized mitophagy pathway is the PINK1/Parkin axis. Under basal conditions, PINK1 is imported into healthy mitochondria and rapidly degraded. When mitochondria lose membrane potential, PINK1 accumulates on the outer mitochondrial membrane and recruits the E3 ubiquitin ligase Parkin, initiating ubiquitination of mitochondrial proteins and the subsequent engulfment of the damaged organelle by the autophagy machinery [120,121]. Recent experimental studies further demonstrate that urolithin A enhances mitochondrial quality control and attenuates age-related functional decline in preclinical models, supporting its role as a potent modulator of mitophagy and mitochondrial homeostasis [122]. Aging impairs this pathway, reducing the cell’s ability to clear dysfunctional mitochondria efficiently. As a result, damaged organelles accumulate, ATP generation declines, ROS production rises, and senescence-associated phenotypes intensify [110,120].

This age-related decline in basal mitophagy is not a trivial secondary event. Recent work suggests that suppressed basal mitophagy itself can be a driver of cellular aging, rather than merely a marker of it [123]. In other words, once mitophagic flux falls below a critical threshold, damaged mitochondria begin to accumulate at a rate that overwhelms compensatory systems, leading to persistent redox stress and altered inflammatory outputs. This concept is especially relevant for a polyphenol-centered review because it reframes mitochondrial protection: the most effective compounds may not simply reduce ROS acutely, but instead restore the cellular decision architecture that determines whether damaged mitochondria are repaired, segregated, or eliminated.

### 6.4. Urolithin A and the Proof-of-Concept for Diet–Microbiome–Mitophagy Signaling

A particularly important development in this field is the emergence of urolithin A as a translational proof-of-concept for mitochondrial quality control. Urolithin A is not a parent dietary polyphenol but a gut microbial metabolite derived from ellagitannins and ellagic acid [32,124]. It is mechanistically important because it demonstrates that the anti-aging impact of polyphenol-rich diets may depend substantially on host–microbiome biotransformation. Preclinical work established urolithin A as a mitophagy inducer capable of improving mitochondrial function and muscle-related outcomes, and more recent human studies have extended this concept [32,79,80]. In randomized placebo-controlled trials, oral urolithin A increased mitochondrial gene signatures and has shown measurable effects on age-related immune and mitochondrial phenotypes in adults, supporting the idea that restoration of mitophagy is clinically actionable [79,80,125].

The importance of urolithin A in the present manuscript is not that it replaces the parent polyphenols discussed earlier, but that it clarifies the mechanism by which dietary polyphenol exposure can be translated into mitochondrial benefit. It shifts the discussion from “polyphenols as direct antioxidants” toward a more biologically realistic framework in which diet, microbial metabolism, mitochondrial turnover, and organismal aging are mechanistically linked. This is particularly valuable for aging biology because it explains why inter-individual responses to polyphenol-rich diets are often heterogeneous: not all individuals generate the same metabolite profile, and not all mitochondrial phenotypes respond identically to enhanced mitophagy [29,30,31,32].

### 6.5. ROS, Mitohormesis, and the Need for Mechanistic Nuance

A scientifically rigorous discussion of polyphenols and mitochondria must also avoid an overly simplistic view of ROS. Mitochondrial ROS are not exclusively harmful; at physiological levels, they serve signaling functions that contribute to adaptation, redox communication, and stress resilience [126]. This concept is closely related to mitohormesis, whereby mild stress induced by compounds such as polyphenols may trigger adaptive mitochondrial responses that enhance cellular resilience and stress resistance [11]. The problem in aging is not ROS per se, but persistent dysregulation of ROS generation relative to detoxification and repair capacity [113,126]. Recent evidence also suggests that the timing and dynamics of mitophagy are critical determinants of cellular aging outcomes, indicating that early activation of mitochondrial turnover pathways may prevent the accumulation of dysfunctional organelles [127]. This distinction is important because many polyphenols are still casually described as antioxidants, when their more biologically meaningful action may be the recalibration of redox-sensitive signaling networks such as Nrf2, AMPK, and NF-κB [83,126]. In some settings, mild stress signaling induced by polyphenols may even promote adaptive responses resembling mitohormesis [128]. Thus, the anti-aging role of polyphenols should be understood less as indiscriminate ROS suppression and more as restoration of redox competence and mitochondrial signaling fidelity.

### 6.6. Mechanistic Convergence: Why Mitochondrial Quality Control Sits at the Center

Taken together, the evidence suggests that mitochondrial quality control is one of the most plausible mechanistic convergence points through which polyphenols influence aging [110,114]. AMPK and SIRT1 promote mitochondrial biogenesis and metabolic adaptation; mTOR inhibition facilitates autophagic turnover; Nrf2 preserves the redox environment required for mitochondrial integrity; and NF-κB suppression prevents chronic inflammatory amplification that otherwise worsens mitochondrial injury [81,82,83,84]. These pathways do not operate in isolation. Their biologically relevant convergence is the maintenance of a mitochondrial population that is energetically competent, redox-balanced, and sufficiently dynamic to undergo renewal. When polyphenols are reported to support this convergence, the associated outcomes may include reduced oxidative damage, attenuation of senescence-associated signaling, and improved metabolic flexibility. Importantly, these effects should not be interpreted as direct or uniform outcomes, but rather as context-dependent processes influenced by cellular state, metabolic conditions, and organismal variability. For this reason, mitochondrial quality control should be viewed not simply as one outcome among many, but as a central mechanistic bridge between polyphenol signaling and healthy aging.

## 7. Polyphenols and the Gut Microbiome in Aging

**Figure 4:**
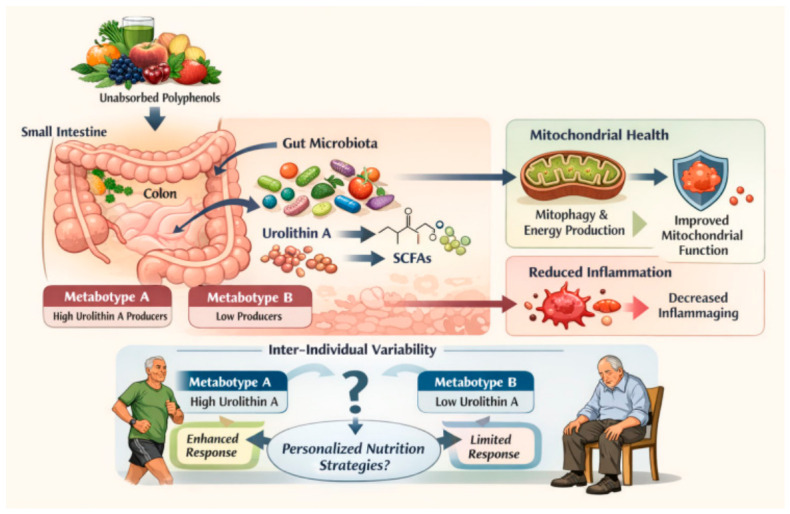
Gut microbiome-mediated transformation of dietary polyphenols and its role in mitochondrial regulation and aging. A substantial fraction of dietary polyphenols escapes absorption in the small intestine and reaches the colon, where it undergoes extensive biotransformation by gut microbiota. Through enzymatic processes, intestinal microbes convert polyphenols into bioactive metabolites, including urolithin A and short-chain fatty acids (SCFAs), which exhibit enhanced bioavailability and biological activity compared with their parent compounds. Urolithin A, a representative microbiota-derived metabolite, induces PINK1/Parkin-dependent mitophagy, thereby promoting the selective removal of dysfunctional mitochondria and improving mitochondrial quality control. In parallel, SCFAs regulate immune responses, intestinal barrier integrity, and metabolic signaling pathways, indirectly influencing mitochondrial function and inflammatory status. Polyphenols also modulate gut microbial composition by promoting beneficial taxa, such as *Bifidobacterium*, *Lactobacillus*, and *Akkermansia*, while suppressing pro-inflammatory microbial species. Importantly, inter-individual variability in microbiome composition leads to distinct metabotypes, which determine the capacity to generate bioactive metabolites and influence responsiveness to polyphenol intake. Collectively, these interactions establish the gut microbiome as a critical mediator linking dietary polyphenols to mitochondrial function, systemic homeostasis, and aging-related physiological outcomes.

In recent years, the gut microbiome has emerged as a key regulator of host physiology and aging [129,130,131,132]. The human gastrointestinal tract contains trillions of microorganisms that participate in numerous metabolic, immune, and endocrine processes. These microbial communities influence host metabolism, immune system maturation, nutrient absorption, and barrier integrity [130,131,132,133]. Increasing evidence indicates that age-associated alterations in microbiota composition—commonly referred to as age-related dysbiosis—play a significant role in the progression of aging and age-related diseases [131,132,133,134]. To better understand how polyphenols interact with the gut microbiome in the context of aging, it is necessary to first consider age-related changes in microbial composition, followed by the microbial metabolism of polyphenols, the generation of bioactive metabolites, and their downstream effects on mitochondrial function and systemic physiology [Figure 4].

### 7.1. Age-Related Microbiome Changes and Inflammaging

Aging is typically associated with reduced microbial diversity, shifts in microbial community structure, and an increased abundance of pro-inflammatory microbial taxa [131,132,133,134,135]. These changes can compromise intestinal barrier integrity and promote the translocation of microbial components such as lipopolysaccharides (LPS) into systemic circulation [134,135,136]. The resulting immune activation contributes to chronic low-grade inflammation, commonly referred to as inflammaging [10,17,134]. Importantly, inflammaging is closely linked to mitochondrial dysfunction, metabolic dysregulation, and cellular senescence [110,135]. Persistent inflammatory signaling can impair mitochondrial function, increase reactive oxygen species (ROS) production, and promote senescence-associated pathways [93,99,110]. Therefore, age-related dysbiosis is not merely a consequence of aging but may actively contribute to multiple hallmarks of aging through systemic inflammatory and metabolic effects.

### 7.2. Microbial Metabolism of Polyphenols and Bioactive Metabolites

Dietary polyphenols interact extensively with the gut microbiome [136,137,138]. Although polyphenols are abundant in plant-based foods, many exhibit limited bioavailability in the upper gastrointestinal tract. After ingestion, a substantial fraction escapes absorption in the small intestine and reaches the colon, where it undergoes extensive microbial metabolism [136,137]. Through enzymatic processes such as deglycosylation, dehydroxylation, and ring cleavage, intestinal microbes convert polyphenols into smaller metabolites with enhanced bioavailability and biological activity [137,138,139]. In many cases, these metabolites, rather than the parent compounds, represent the primary bioactive forms responsible for systemic effects. Thus, the physiological impact of polyphenols is strongly dependent on host–microbiome interactions [138,139]. Recent evidence further supports that microbiome-derived metabolites such as urolithin A play a central role in mediating the systemic effects of dietary polyphenols, with inter-individual variability driven by gut microbial composition [124] (Kuerec et al., 2024). One of the most well-characterized examples is urolithin A, a metabolite derived from ellagitannins and ellagic acid present in foods such as pomegranates, walnuts, and berries [79,139,140]. Urolithin A has attracted significant attention because it acts as a potent inducer of mitophagy, facilitating the selective removal of dysfunctional mitochondria [32,124]. Experimental studies have demonstrated that urolithin A enhances mitochondrial respiratory capacity and improves muscle function in aging models [124,140]. Furthermore, clinical studies in older adults have shown that supplementation with urolithin A increases mitochondrial gene expression signatures associated with improved mitochondrial health [79,80]. In addition to urolithin A, microbial fermentation processes generate short-chain fatty acids (SCFAs), including acetate, propionate, and butyrate [141]. These metabolites act as key signaling molecules that regulate immune responses, intestinal barrier function, and metabolic homeostasis [141,142,143]. SCFAs have also been implicated in the modulation of mitochondrial metabolism and inflammatory signaling, further linking microbiome activity to aging-related cellular processes [142,143]. Importantly, this interaction is bidirectional, as mitochondrial function can influence host metabolic and immune environments, which in turn shape gut microbiome composition and activity [144].

### 7.3. Microbiome Remodeling and Systemic Effects

Beyond metabolite production, polyphenols can actively reshape the composition and function of gut microbial communities [138,145]. Several studies have reported that polyphenol-rich diets promote the growth of beneficial microbial taxa such as *Bifidobacterium*, *Lactobacillus*, and *Akkermansia*, while suppressing pathogenic or pro-inflammatory species [145,146,147]. These compositional changes can enhance intestinal barrier integrity, improve mucosal immunity, and reduce systemic inflammation [134,145]. Through these effects, polyphenols may indirectly influence host physiology at multiple levels, including metabolic regulation, immune modulation, and mitochondrial function. In addition, microbiota-derived metabolites can enter systemic circulation and influence distant organs, contributing to the regulation of the gut–brain axis and neuroinflammatory pathways [148,149]. This highlights the potential role of polyphenol–microbiome interactions in modulating neurodegenerative processes associated with aging.

### 7.4. Inter-Individual Variability and Translational Challenges

Despite increasing evidence supporting the role of microbiome-mediated polyphenol metabolism in aging, significant challenges remain. One of the most important limitations is inter-individual variability in microbiome composition, which influences the capacity to generate specific bioactive metabolites [138,139,150]. For example, only certain individuals harbor microbial communities capable of producing urolithin A, leading to the classification of distinct metabotypes [139,150]. This variability may explain the heterogeneous responses observed in clinical studies of polyphenol interventions. In addition, microbial metabolic networks are highly complex, involving multiple species and pathways that can generate diverse metabolites with different biological activities [137,151]. Consequently, the systemic effects of polyphenols cannot be fully understood without integrating microbiome composition, metabolic profiling, and host physiological responses [151]. Future research should therefore adopt a systems-level approach combining microbiome analysis, metabolomics, and molecular biology to better characterize polyphenol–microbiome interactions. Such approaches may facilitate the development of personalized nutritional strategies aimed at promoting healthy aging.

## 8. Therapeutic Potential of Polyphenols in Age-Related Diseases

**Figure 5:**
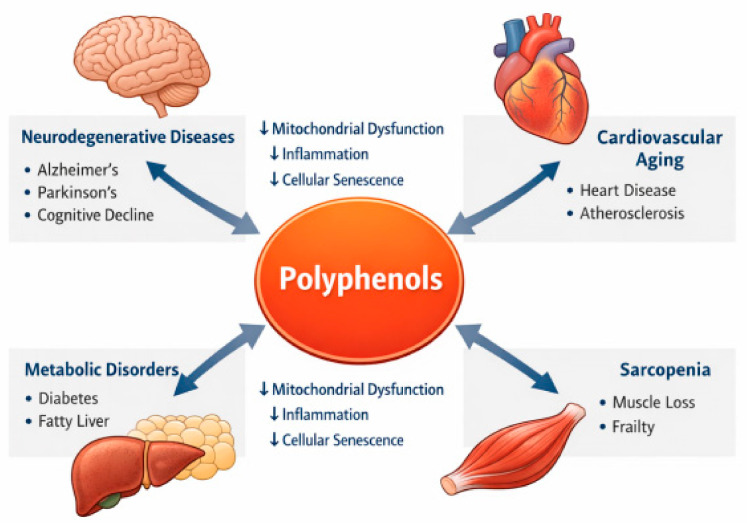
Polyphenols as modulators of shared pathogenic mechanisms across age-related diseases. Polyphenols exert pleiotropic effects on multiple age-related diseases by targeting common underlying biological processes rather than disease-specific pathways. Major classes of polyphenols influence conserved signaling networks, including AMP-activated protein kinase (AMPK), sirtuins (SIRT1), mechanistic target of rapamycin (mTOR), nuclear factor erythroid 2–related factor 2 (Nrf2), and nuclear factor-κB (NF-κB), which regulate mitochondrial function, redox balance, inflammation, and cellular senescence. Through coordinated modulation of these pathways, polyphenols are associated with reduced mitochondrial dysfunction, attenuation of chronic inflammation (inflammaging), and suppression of senescence-associated signaling. These effects contribute to improved cellular and tissue homeostasis across multiple organ systems, including the brain (neurodegenerative diseases), cardiovascular system (vascular aging and heart disease), metabolic tissues (metabolic disorders), and skeletal muscle (sarcopenia and frailty). Collectively, these observations support the concept that polyphenols act as systems-level modulators of aging biology, targeting shared mechanistic drivers of age-related multimorbidity.

The translational relevance of polyphenols in aging research is based on a key biological premise [152,153,154]. Most age-related diseases do not arise from a single isolated lesion but from the progressive interaction of mitochondrial dysfunction, chronic inflammation, proteostatic failure, altered nutrient sensing, and cellular senescence [152,153,154]. Consequently, compounds that modulate several of these processes simultaneously may be better suited to age-related multimorbidity than highly specific single-target interventions [152,153,154,155]. In this context, polyphenols are particularly attractive because they repeatedly converge on conserved regulatory nodes such as AMPK, SIRT1, mTOR, Nrf2, and NF-κB, while also affecting autophagy, mitochondrial turnover, and microbiota-derived signaling [155,156]. This multi-target profile is mechanistically aligned with the systems biology of aging [152,155]. It contrasts with the reductionist pharmacology of single-disease treatment. However, the therapeutic promise of polyphenols should not be overstated [153,156]. Their translational value does not necessarily lie in replacing established drugs. Instead, they may function as geroprotective modulators that reduce biological vulnerability, attenuate disease progression, and improve tissue resilience under aging-associated stress [153,156]. This distinction is crucial because many preclinical studies demonstrate improvements in biomarkers, mitochondrial function, inflammatory tone, or proteostatic balance without necessarily showing disease reversal [153,156]. Therefore, the most scientifically defensible view is that polyphenols may be useful as adjunctive or preventive interventions within broader anti-aging strategies, especially where mitochondrial dysfunction, inflammaging, or senescence are central pathogenic drivers [152,153,154,155,156]. These findings highlight the potential of polyphenols as geroprotective modulators targeting shared biological mechanisms across diverse age-related diseases in Figure 5.

### 8.1. Neurodegenerative Diseases

Neurodegenerative diseases represent one of the clearest settings in which polyphenol-based interventions may have biological plausibility [157,158,159,160]. Aging is the major risk factor for disorders such as Alzheimer’s disease and Parkinson’s disease, and the underlying pathology in both conditions is tightly linked to mitochondrial dysfunction, oxidative stress, chronic neuroinflammation, defective proteostasis, and impaired autophagy [157,158,159,160]. Neurons are especially vulnerable to these processes [158,159]. They are post-mitotic, highly energy-dependent, and rely on efficient mitochondrial turnover to maintain synaptic transmission and calcium handling [158,159]. As a result, interventions that preserve mitochondrial quality control and suppress inflammatory amplification are mechanistically relevant in neurodegenerative aging [157,158,159,160].

Curcumin, resveratrol, and quercetin have each been implicated in neuroprotective mechanisms that are relevant to this context [160,161,162,163,164]. Curcumin has been reported to improve autophagy-related signaling in experimental Alzheimer’s models through AMPK/JNK-associated suppression of mTOR and modulation of LC3 and p62, suggesting that its effects extend beyond generic anti-inflammatory activity into proteostatic and degradative processes [160,161]. Resveratrol has been linked to enhanced mitophagy and reduced amyloid- or α-synuclein-associated toxicity through SIRT1-related signaling [162,163], while quercetin has been associated with SIRT1/FOXO1/NRF2 modulation, reduced neuronal stress, and improved autophagic handling of toxic aggregates [164]. These findings suggest that polyphenols may influence multiple pathogenic layers simultaneously. These include mitochondrial dysfunction, aggregate clearance, glial inflammation, and neuronal survival [160,161,162,163,164].

From a disease-mechanism perspective, this pleiotropy may be particularly useful in Alzheimer’s disease, where amyloid burden alone does not explain the full extent of synaptic failure and cognitive decline [157,158,159]. Aging neurons experience mitochondrial depolarization, reduced ATP generation, impaired mitophagy, and elevated ROS even before severe structural neurodegeneration becomes evident [158,159]. In such a setting, a compound that supports autophagy, stabilizes mitochondrial function, and suppresses inflammatory transcription may exert cumulative benefit even if its effect on a single lesion is modest. Similarly, in Parkinson’s disease, where mitochondrial dysfunction and defective PINK1/Parkin-related quality control are central to dopaminergic vulnerability, polyphenols that reinforce mitochondrial maintenance may be especially relevant [157,159,162].

The role of urolithin A strengthens this translational argument. Urolithin A has shown neuroprotective and brain aging-related benefits in experimental systems and improves mitochondrial programs through mitophagy-related mechanisms [165,166,167]. Its importance here is conceptual as much as therapeutic: it demonstrates that the anti-neurodegenerative potential of polyphenol-rich diets may depend substantially on microbial conversion into metabolites with better bioactivity and mitochondrial specificity than the parent compounds [165,166,167]. This provides a bridge between neurodegeneration, mitochondrial quality control, and the gut–brain axis.

At the translational level, the main limitation is that promising mechanistic and preclinical findings have not yet translated into unequivocal clinical efficacy [157,160]. Bioavailability, blood–brain barrier penetration, dose selection, and inter-individual metabolic variability all complicate interpretation [157,160]. Nonetheless, the available evidence supports the view that polyphenols are not merely symptomatic antioxidants, but potential modulators of the core stress-adaptation architecture that becomes destabilized in neurodegenerative aging [157,160].

### 8.2. Cardiovascular Aging

Cardiovascular aging is characterized by endothelial dysfunction, vascular stiffening, altered cardiac energetics, chronic inflammatory activation, and impaired autophagy/mitophagy in long-lived post-mitotic cells such as cardiomyocytes [168,169,170,171]. These cells depend heavily on mitochondrial integrity and basal autophagic flux [169,170,171]. Age-associated suppression of these systems promotes hypertrophic remodeling, increased oxidative burden, ischemic vulnerability, and progressive functional decline [168,169,170,171]. This makes the cardiovascular system particularly relevant for polyphenol research, since many polyphenols influence AMPK/mTOR, SIRT1/FoxO, and redox-sensitive pathways that are directly linked to cardiomyocyte survival and vascular homeostasis [169,170,171,172,173].

Curcumin has been shown in cardiovascular models to enhance autophagic flux, improve the expression of Beclin-1 and ATG-related proteins, and attenuate oxidative stress and cardiomyocyte senescence [172,173]. These findings suggest that curcumin may preserve cardiac homeostasis not only through anti-inflammatory action, but also by restoring degradative capacity in stressed myocardium [172,173]. Resveratrol, in turn, has been linked to increased LC3 and Beclin-1, improved mitochondrial ATP production, and reduced endothelial oxidative damage through SIRT1- and TFEB-associated pathways [171,173,174]. Quercetin has similarly been associated with AMPK-mediated increases in LC3 and Beclin-1, attenuation of myocardial injury, and beneficial effects in oxidative and ischemic cardiovascular contexts [173,175]. Together, these observations suggest that the cardiovascular benefits of polyphenols are strongly tied to the restoration of cellular housekeeping systems rather than to one-dimensional vasodilatory or antioxidant effects.

This mechanistic framing is important because cardiovascular aging is not simply a disease of “too much ROS” or “too much inflammation”. Rather, it is a syndrome of declining mitochondrial adaptability, inadequate organelle clearance, impaired nutrient-sensing responses, and chronic inflammatory reinforcement [168,169,170,171]. Polyphenols are therefore potentially useful because they touch several of these layers simultaneously. For example, AMPK activation can improve metabolic efficiency and autophagic responsiveness, SIRT1 can favor endothelial resilience and mitochondrial adaptation, and NF-κB suppression can reduce chronic vascular inflammation [169,170,171,172,173,174]. When these changes occur together, the expected benefit is not merely lower oxidative injury, but better preservation of the aged cardiovascular phenotype under stress.

Nonetheless, clinical translation in cardiovascular aging remains incomplete. The strongest mechanistic evidence still comes from preclinical and translationally proximal models rather than from large outcome trials in older populations [168,171]. This means that the current role of polyphenols is better justified as a mechanistically plausible adjuvant strategy than as a stand-alone cardioprotective therapy.

### 8.3. Metabolic Disorders and Metabolic Aging

Metabolic disorders are among the most prominent age-associated conditions and include insulin resistance, obesity, type 2 diabetes, dyslipidemia, and metabolic dysfunction–associated fatty liver disease [176,177,178,179]. These disorders are not only consequences of aging but also active drivers of biological aging [176,177,178,179]. They amplify oxidative stress, mitochondrial dysfunction, low-grade inflammation, and cellular senescence [176,177,178,179]. In this respect, metabolic disease and aging exist in a bidirectional relationship: aging promotes metabolic inflexibility, and metabolic dysfunction feeds back to worsen aging hallmarks [176,177,178,179].

Polyphenols are particularly relevant in this domain because many of them target the energy-sensing architecture that becomes dysregulated during metabolic aging [155,177,180]. Resveratrol, for example, has been associated with improved skeletal muscle mitochondrial function in older adults, with attenuation of sarcopenic-obesity-associated mitochondrial dysfunction in rodents, and with modulation of glucose and lipid metabolism through AMPK/SIRT1-linked signaling [180,181]. Curcumin has shown benefits in metabolic syndrome-related parameters in both experimental and clinical contexts [182], while EGCG and related green tea polyphenols have been associated with AMPK activation, greater fatty acid oxidation, suppression of lipid synthesis, and reduced obesity-related inflammatory burden [183]. The mechanistic importance of this should not be reduced to “better metabolism”. At a deeper level, polyphenols may restore the coupling between nutrient sensing and maintenance programs [155,177,180]. In metabolic aging, chronic caloric excess and insulin resistance drive persistent mTOR activation, suppress autophagy, impair mitochondrial renewal, and intensify inflammatory signaling [176,177,178,179]. Polyphenols that activate AMPK, stimulate SIRT pathways, or attenuate nutrient-overload signaling may therefore partly re-establish a more youthful balance between growth and repair. This is why some polyphenols are described as caloric restriction mimetics or nutrient-sensing modulators rather than simply as metabolic supplements [180,181].

Quercetin also occupies an important place in metabolic aging because its senolytic/senomorphic profile intersects with adipose tissue inflammation and insulin resistance [184]. In older models, dasatinib plus quercetin has been associated with reduced inflammatory gene expression, improved systemic metabolic function, and attenuation of intestinal senescence and barrier dysfunction [184,185]. These observations suggest that the metabolic benefit of quercetin may extend beyond direct intracellular signaling and include a reduction of senescence-driven inflammatory amplification across metabolically active tissues.

For metabolic disorders, the translational rationale for polyphenols is therefore particularly strong: the target biology is systemic, modifiable, and mechanistically aligned with pathways that polyphenols are already known to regulate [155,180,181,182,183,184]. The limitation, again, is that durable clinical efficacy, optimal dose, formulation, and responder stratification remain incompletely defined [182,183].

### 8.4. Sarcopenia, Frailty, and Musculoskeletal Aging

Sarcopenia and frailty are clinically important manifestations of aging [186]. They reflect the combined effects of mitochondrial dysfunction, chronic inflammation, neuromuscular decline, and reduced regenerative capacity [186,187]. Skeletal muscle is one of the most metabolically active tissues in the body and is highly dependent on mitochondrial function for endurance, anabolic responsiveness, and stress adaptation [181,182]. With aging, mitochondrial biogenesis decreases, mitophagy becomes less efficient, redox balance deteriorates, and anabolic signaling becomes less responsive, all of which contribute to loss of muscle quality and functional decline [186,187].

Resveratrol has shown some of the strongest aging-related muscle evidence among the major polyphenols. In older adults with functional limitations, it has been associated with improved skeletal muscle mitochondrial function and physical-function-related parameters, while in animal models it has ameliorated mitochondrial dysfunction, oxidative stress, and altered protein metabolism linked to sarcopenic obesity [181,188]. These findings are mechanistically coherent with its effects on SIRT1, AMPK, mitochondrial biogenesis, and autophagic renewal [181,188].

The relevance of urolithin A is especially strong in this domain. Preclinical and clinical evidence indicates that urolithin A enhances mitophagy, improves mitochondrial respiratory signatures, and supports muscle-related mitochondrial health in aging [165,167,189]. This is particularly important for sarcopenia because it positions mitochondrial quality control as a modifiable driver of functional decline rather than as an inevitable correlate of old age. In other words, the muscle-aging field provides one of the clearest examples in which a polyphenol-derived metabolite has moved from mechanistic plausibility to early human translational promise [165,167,189].

From a broader geroscience standpoint, the significance of these findings is that they link dietary patterns, microbiome metabolism, mitochondrial renewal, and physical function into a unified translational framework [165,189]. Because frailty is often the emergent phenotype of multisystem aging, compounds that improve mitochondrial resilience in muscle may also influence broader healthspan trajectories [186,187].

### 8.5. Translational Interpretation

Taken together, evidence across neurodegenerative, cardiovascular, metabolic, and musculoskeletal aging supports a common conclusion [152,153,154,155,156]. The therapeutic potential of polyphenols lies not in disease-specific effects but in their ability to modulate shared pathogenic processes across multiple age-related conditions [152,155,156]. These shared processes include defective autophagy, impaired mitochondrial quality control, chronic inflammatory activation, oxidative imbalance, and senescence-associated tissue deterioration [152,153,154,155,156]. Because age-related diseases arise from repeated failure of these same maintenance systems in different tissues, polyphenols are biologically attractive precisely because they engage these common nodes [152,155]. At the same time, scientific rigor requires acknowledging that preclinical richness has outpaced clinical certainty [153,156]. The next translational step is not simply to run more trials, but to design better ones—incorporating pharmacokinetics, metabolite profiling, microbiome stratification, mitochondrial biomarkers, and aging-relevant functional endpoints [156,165,189]. Only then will it become possible to determine whether polyphenols are best deployed as preventive dietary factors, targeted nutraceuticals, adjunctive gerotherapeutics, or personalized microbiome-dependent interventions.

## 9. Limitations, Challenges, and Future Perspectives

Despite extensive mechanistic evidence supporting the anti-aging potential of polyphenols, several major limitations hinder their translation into effective geroprotective interventions [190,191]. A key issue is that biological effects observed in vitro are often difficult to extrapolate to human physiology [190,191,192]. In many in vitro studies, polyphenols are applied at concentrations ranging from approximately 10–100 μM or higher, whereas plasma concentrations following dietary intake are typically in the nanomolar to low micromolar range. For example, circulating levels of most polyphenols and their metabolites in humans generally remain below 1–2 μM, often in the nanomolar range, due to limited absorption and rapid metabolism. Moreover, the bioavailability of many dietary polyphenols is relatively low (commonly <5–10%), and most circulating forms consist of conjugated metabolites rather than parent compounds. These discrepancies highlight a critical gap between experimental conditions and physiological relevance and underscore the need for careful interpretation when extrapolating in vitro findings to in vivo or clinical contexts [192,193,194,195,196]. Many studies use concentrations that are not achievable through dietary intake, and circulating forms in vivo frequently consist of conjugated metabolites rather than the parent compounds [190,196]. Consequently, the molecular species responsible for biological activity in humans may differ substantially from those examined in experimental systems [190,196].

Bioavailability and metabolism represent additional challenges [185,186,187,188]. Polyphenols vary widely in absorption, distribution, and elimination, and their biological effects are strongly influenced by host metabolism and gut microbiota [185,188]. In many cases, microbiota-derived metabolites, rather than the original compounds, appear to be the primary bioactive forms [139,165,197]. This is exemplified by urolithin A, highlighting the importance of host–microbiome interactions [139,165]. Inter-individual variability in microbiome composition further contributes to heterogeneous responses, emphasizing the need for stratification of participants in future studies [150,197]. Another limitation is the reliance on surrogate biomarkers. While changes in oxidative stress, inflammation, or mitochondrial signaling are informative, they do not necessarily translate into clinically meaningful improvements in healthspan [198]. Future studies should therefore incorporate integrated outcome measures, including functional, physiological, and longitudinal endpoints [198,199].

Clinical research in this field also remains limited by small sample sizes, short intervention durations, and heterogeneous study designs [191,198]. Improved trial design is needed, including standardized formulations, pharmacokinetic and metabolite profiling, validated aging-related biomarkers, and appropriate participant selection based on relevant biological phenotypes such as mitochondrial dysfunction or inflammaging [191,198,199]. Importantly, polyphenols should not be viewed simply as antioxidants [200]. Their biological effects are better understood as modulation of interconnected signaling networks, including nutrient sensing, redox regulation, autophagy, and inflammatory pathways [200,201]. In addition, dose–response relationships and context dependence remain insufficiently characterized, and polyphenols may exhibit non-linear or hormetic effects [200,202]. These discrepancies highlight the need for improved integration of pharmacokinetic and pharmacodynamic considerations when interpreting the biological effects of polyphenols. Future research should adopt systems-level approaches integrating multi-omics technologies, microbiome analysis, and clinical phenotyping [199,203]. Such strategies may help identify responsive subgroups, define active metabolites, and clarify mechanisms of action in vivo [197,199,203]. In this context, polyphenols may be most effectively utilized as part of precision nutrition or multimodal interventions rather than as stand-alone therapies [197,204].

Overall, advancing polyphenol-based geroscience will require a shift from descriptive evidence toward mechanistically grounded and clinically integrated research frameworks [191,199]. Addressing these challenges will be essential for determining how polyphenols can be effectively applied to promote healthy aging. It is also important to acknowledge that not all studies report consistent or beneficial effects of polyphenols. Variability in experimental design, dosage, bioavailability, and individual biological responses may lead to heterogeneous or even contradictory outcomes across studies.

## 10. Conclusions

Aging is a multifactorial biological process driven by the progressive dysregulation of molecular pathways that govern cellular homeostasis, stress responses, and metabolic balance [8]. The framework of the hallmarks of aging, including genomic instability, mitochondrial dysfunction, deregulated nutrient sensing, and cellular senescence, has provided a mechanistic foundation for understanding how aging contributes to chronic disease development and functional decline across multiple tissues [8].

Within this context, dietary polyphenols have emerged as promising modulators of aging-associated pathways because they influence multiple regulatory networks simultaneously rather than acting through a single molecular target [155]. Evidence indicates that polyphenols regulate key signaling pathways, including AMPK, SIRT1, mTOR, Nrf2, and NF-κB, which coordinate metabolic adaptation, redox balance, autophagy, and inflammatory responses [155]. Through coordinated regulation of these pathways, polyphenols have been shown to influence several hallmarks of aging, including mitochondrial dysfunction, cellular senescence, and chronic inflammation [155].

A central theme emerging from current evidence is the role of mitochondrial quality control as a key mechanistic convergence point in aging biology [110]. Polyphenols such as resveratrol, EGCG, curcumin, and quercetin have been reported to support mitochondrial biogenesis, enhance autophagy and mitophagy, and maintain redox homeostasis through AMPK–SIRT1–PGC-1α–associated signaling networks [102,103]. By preserving mitochondrial function and limiting oxidative damage, these compounds may contribute to improved cellular resilience and delayed progression of aging-related pathologies [110].

In addition, polyphenols influence cellular senescence and inflammaging through modulation of oxidative stress and inflammatory signaling pathways [93]. Accumulation of senescent cells and SASP-associated factors represents a major driver of tissue dysfunction during aging [93]. Polyphenols may attenuate these processes by suppressing inflammatory transcriptional programs and enhancing cellular stress responses [155]. In certain contexts, compounds such as quercetin may also exhibit senotherapeutic effects by targeting survival pathways in senescent cells [107]. Importantly, the biological effects of polyphenols extend beyond direct intracellular signaling. Increasing evidence highlights the critical role of the gut microbiome in mediating the systemic effects of dietary polyphenols [129,138]. Microbial biotransformation generates metabolites, such as urolithin A, with enhanced bioavailability and mitochondrial specificity [165]. These microbiota-derived metabolites link dietary exposure to mitochondrial function, inflammatory regulation, and metabolic homeostasis, providing a systems-level framework for understanding polyphenol activity in aging [138].

Despite substantial mechanistic advances, several challenges remain in translating polyphenol research into effective geroprotective interventions [190,191]. Variability in bioavailability, inter-individual differences in microbiome composition, and the complexity of aging biology contribute to heterogeneous responses observed in clinical studies [190,197]. Future research should therefore focus on improving pharmacokinetic characterization, identifying biomarkers of responsiveness, and integrating multi-omics approaches to better define the systemic effects of polyphenols in aging populations [199].

In conclusion, current evidence supports the view that polyphenols represent a biologically plausible class of geroprotective compounds capable of modulating multiple hallmarks of aging [155]. Rather than functioning solely as direct antioxidants, these compounds act as regulators of interconnected signaling networks that govern mitochondrial function, metabolic adaptation, inflammatory responses, and cellular senescence [200]. Continued interdisciplinary research integrating molecular biology, nutrition science, microbiome research, and clinical investigation will be essential to determine how polyphenols can be effectively incorporated into strategies aimed at extending human healthspan [204].

## Figures and Tables

**Table 1:** Summary of major dietary polyphenols, their molecular targets, and roles in aging-related mechanisms and mitochondrial function.

Compound	Core Targets	Aging-Related Mechanisms	Hallmarks Relevance	Mitochondrial Relevance	Evidence Level	Key Limitations	Ref.
Resveratrol	SIRT1, AMPK, mTOR	Activates SIRT1–PGC-1α signaling and autophagy	Mitochondrial dysfunction; nutrient sensing; inflammaging	Biogenesis, mitophagy	Animal + Human	Low bioavailability	[25,54,55,56]
Pterostilbene	SIRT1, AMPK	Modulates oxidative stress–related pathways	Oxidative stress; mitochondrial aging	Mitochondrial protection	Animal	Limited human data	[25,65]
EGCG	AMPK, Nrf2, NF-κB	Regulates antioxidant and inflammatory signaling	Oxidative stress; inflammation; mitochondrial dysfunction	Redox regulation	Cell + Animal	Stability issues	[26,57,58,59,60]
Catechin	Nrf2	Supports antioxidant defense	Oxidative stress	Redox homeostasis	Cell	Limited evidence	[57,58]
Epicatechin	eNOS, AMPK	Improves vascular and mitochondrial function	Vascular aging	Mitochondrial respiration	Human + Animal	Dose variability	[66]
Curcumin	NF-κB, Nrf2, AMPK, mTOR	Modulates inflammation and autophagy	Inflammaging; oxidative stress	Autophagy-mediated turnover	Animal + Human	Poor bioavailability	[27,61,62]
Quercetin	PI3K/Akt, mTOR, NF-κB	Senolytic/senomorphic activity	Cellular senescence; SASP	Mitochondrial regulation	Animal + Human	Variability	[28,63,64]
Fisetin	PI3K/Akt	Senotherapeutic effects	Senescence; inflammaging	Indirect mitochondrial	Animal	Limited clinical data	[67]
Kaempferol	AMPK, Nrf2	Antioxidant and autophagy modulation	Oxidative stress	Mitochondrial protection	Cell + Animal	Limited in vivo data	[68]
Luteolin	SIRT1, Nrf2	Neuroinflammatory regulation	Brain aging	Neuronal mitochondria	Animal	Limited human data	[69]
Apigenin	FOXO	Lifespan signaling	Stress resistance	Indirect mitochondrial	Model organisms	Limited mammalian evidence	[70]
Naringenin	AMPK	Metabolic regulation	Metabolic aging	Mitochondrial metabolism	Animal	Limited human data	[71]
Naringin	FOXO	Lifespan modulation	Aging pathways	Indirect mitochondrial	Model organisms	Non-mammalian evidence	[72]
Anthocyanins	Nrf2, AMPK	Vascular and oxidative regulation	Vascular aging	Mitochondrial protection	Human + Animal	Heterogeneity	[73]
Chlorogenic acid	AMPK	Glucose/lipid metabolism	Metabolic aging	Mitochondrial metabolism	Animal + Human	Variable metabolism	[74]
Ellagic acid	PPAR-γ	Anti-inflammatory effects	Inflammaging	Microbiome-dependent	Animal	Microbiome variability	[75]
Oleuropein	AMPK, mTOR	Autophagy regulation	Autophagy decline	Mitochondrial turnover	Animal	Limited evidence	[76]
Hydroxytyrosol	Nrf2, AMPK	Redox signaling	Oxidative stress	Mitochondrial protection	Animal	Limited human data	[76,77]
Rosmarinic acid	Nrf2	Antioxidant pathways	Oxidative stress	Indirect mitochondrial	Cell + Animal	Limited evidence	[78]
Urolithin A	PINK1/Parkin	Mitophagy induction	Mitochondrial dysfunction	Direct mitochondrial QC	Human + Animal	Microbiome-dependent	[32,79,80]

## Data Availability

The original contributions presented in this study are included in the article. Further inquiries can be directed to the corresponding author.

## Abbreviations

The following abbreviations are used in this manuscript:

AMPK	AMP-activated protein kinase
ATP	Adenosine triphosphate
ATG	Autophagy-related gene
CVD	Cardiovascular disease
DNA	Deoxyribonucleic acid
EGCG	Epigallocatechin gallate
eNOS	Endothelial nitric oxide synthase
FOXO	Forkhead box O
HO-1	Heme oxygenase-1
IL	Interleukin
IGF-1	Insulin-like growth factor 1
JNK	c-Jun N-terminal kinase
LPS	Lipopolysaccharide
MAPK	Mitogen-activated protein kinase
MMP	Matrix metalloproteinase
mTOR	Mechanistic target of rapamycin
mTORC1	Mechanistic target of rapamycin complex 1
mTORC2	Mechanistic target of rapamycin complex 2
mtDNA	Mitochondrial DNA
mtROS	Mitochondrial reactive oxygen species
NF-κB	Nuclear factor kappa B– Nuclear factor kappa B
Nrf2	Nuclear factor erythroid 2–related factor 2
PGC-1α	Peroxisome proliferator-activated receptor gamma coactivator 1-alpha
PI3K	Phosphoinositide 3-kinase
Rb	Retinoblastoma protein
ROS	Reactive oxygen species
SASP	Senescence-associated secretory phenotype
SCFA	Short-chain fatty acid
SIRT	Sirtuin
TFEB	Transcription factor EB
TNF-α	Tumor necrosis factor-alpha
ULK1	Unc-51-like kinase 1
